# Physician and patient perceptions of fecal microbiota transplant for recurrent or refractory *Clostridioides difficile* in the first 6 years of a central stool bank

**DOI:** 10.1002/jgh3.12396

**Published:** 2020-08-06

**Authors:** Madeleine Gill, Charlotte Blacketer, Franco Chitti, Karmen Telfer, Lito Papanicolas, Lisa M Dann, Emily C Tucker, Robert V Bryant, Samuel P Costello

**Affiliations:** ^1^ Department of Gastroenterology The Queen Elizabeth Hospital Adelaide South Australia Australia; ^2^ School of Medicine University of Adelaide Adelaide South Australia Australia; ^3^ School of Medicine Flinders University Adelaide South Australia Australia; ^4^ Department of Infectious Diseases Royal Adelaide Hospital Adelaide South Australia Australia; ^5^ BiomeBank Adelaide South Australia Australia; ^6^ Department of Infectious Diseases Flinders Medical Centre Adelaide South Australia Australia

**Keywords:** *Clostridioides difficile*, fecal microbiota transplantation, perception, physician, stool bank

## Abstract

**Background and Aim:**

Fecal microbiota transplantation (FMT) is a highly effective therapy for recurrent or refractory *Clostridioides difficile* infection (rCDI). Despite inclusion in society guidelines, the uptake of FMT therapy has been variable. Physician and patient attitudes may be a barrier to evidence‐based uptake of therapies; however, data assessing attitudes regarding FMT for rCDI are limited.

**Methods:**

The South Australian FMT for CDI database prospectively recorded patient outcomes of FMT for CDI from August 2013 to January 2019. A total of 93 consecutive patients who underwent FMT for rCDI in South Australia were invited to participate in a 20‐question survey regarding the patient experience of FMT. All gastroenterologists and infectious disease physicians practicing in South Australia were invited to participate in an online survey comprised of 22 questions that addressed referral experience, indications for referral, perceived risks, and regulation and funding.

**Results:**

Fifty‐four patients (54/93, 58%) returned the survey, of whom 52 (96%) would recommend FMT to others, and 51 (94%) were satisfied with treatment outcome. Fifty physicians returned the online survey (50/100, 50%), of whom 23 (46%) were concerned about disease transmission risk, and 15 (30%) believed that the risk of FMT would outweigh the benefit. Infectious diseases physicians and advanced trainees had significantly greater concern regarding the potential alteration of the microbiome than gastroenterology physicians and advanced trainees (8/17 (47%) *vs* 6/33 (18%); *P* = 0.047).

**Conclusion:**

Despite high levels of patient‐reported satisfaction following FMT, physician‐reported reservations exist and may present a barrier to uptake of this therapy.

## Graphical abstract

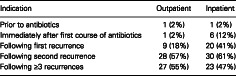

Fecal microbiota transplantation (FMT) is a highly effective therapy for recurrent or refractory *Clostridioides difficile* infection; however, physician and patient attitudes may be a barrier to uptake. Of 54 surveyed FMT recipients, 52 (96%) would recommend FMT treatment to others. In contrast, 15 of 50 (30%) physicians believed the risk of FMT outweighed the benefit.

## Introduction

*Clostridioides difficile* infection (CDI) is the most common cause of health‐care‐associated diarrhea and is associated with significant morbidity, mortality, and costs worldwide.^1^ Recurrence of CDI following standard first‐line antibiotics is common and occurs in approximately 35%.^2^ In those patients who do relapse, further antibiotic treatments give diminishing rates of cure; after a second recurrence, the chance of further recurrence increases to 60% and is even greater for subsequent recurrences.^3^ Fecal microbiota transplantation (FMT), the transfer of stool from a healthy individual to a person with disease, has emerged over the last decade as the most effective therapy for recurrent or refractory CDI (rCDI), with rates of primary cure between 81 and 96%.^4^, ^5^ FMT is well tolerated and safe, with very few serious adverse effects,^4^ even in the elderly and immunocompromised, and is more cost‐effective than traditional antibiotic therapy for rCDI.^6^

Despite a strong body of evidence supporting the efficacy and safety of FMT for CDI and inclusion in treatment guidelines,^7^, ^8^, ^9^, ^10^, ^11^ the uptake of this therapy has been variable, with many centers worldwide still unable to provide the service.^10^ Local logistical and regulatory issues exist, which serve as barriers to widespread and equitable access to FMT for patients with rCDI.^12^ In addition to this, physician and patient attitudes may also be barriers to the uptake of FMT, with limited awareness, provider resistance, and lack of availability cited as potential contributing factors.^13^

With the establishment of a state‐based stool bank in South Australia in 2013,^14^, ^15^ there has been universal access to this therapy within the public health system in the state. However, the degree of patient and physician awareness of, and experience with, FMT is not known. Furthermore, it is unknown whether inconsistencies between patient and physician attitudes toward FMT still exist and if attitudinal factors may affect the uptake of this therapy. The aim of this study was therefore to gain an understanding of patient and physician perception and experience with FMT for CDI in South Australia and identify attitudinal discrepancies to provide insight into potential barriers of uptake of this therapy in the future.

## Methods

### *Study participants*

The South Australian FMT for CDI database was interrogated, and the first 93 consecutive patients to undergo FMT for rCDI in South Australia from August 2013 to January 2019 were included and invited to participate in the survey. Patient surveys were posted and returned via mail.

Both gastroenterologists and gastroenterology trainees, as well as infectious disease (ID) physicians and ID trainees, practicing in public and private hospital systems were identified using existing practitioner registers and contacted via email. These registers contain all known practicing gastroenterology and ID doctors in the state. A total of 69 gastroenterologists and 31 ID physicians from South Australia were invited to participate in an online survey via email.

### *Survey development*

A 20‐item paper survey was devised for the patient group (patient survey questions listed in (Table 1). An electronic survey (Table 2) was developed for the physician group using the online program SurveyMonkey. It was distributed via email in April and May 2018. It comprised 22 items and addressed referral experience, indications for referral, perceived risks, and regulation and funding. The Central Adelaide Local Health Network ethics committee approved the distribution of the surveys and collection of study data.

**Table 1:** Patient survey questions

Question	Options
What is your age?	
What is your gender?	Male Female
What date was your FMT?	
How was your FMT delivered?	Via colonoscopy Via endoscopy Naso‐jejunal tube Enemas
Had you heard of FMT prior to developing *Clostridium difficile* infection?	Yes No
How did you first hear of FMT as a treatment?	Treating specialist General practitioner Media Friend Other
What was your perception of FMT when first discussed with your doctor as a treatment for your *C. difficile*?	I had no concerns, I just wanted to get better I was concerned about: the “yuck” factor infection risk contracting other disease the colonoscopy other
Did this perception change after FMT?	Yes No If yes, how?
Would you recommend FMT to other patients with CDI?	Yes No
How many relapses did you have prior to FMT?	
Have you had a relapse since FMT?	Yes No If yes, how was this treated?
How long did it take for symptoms to resolve after FMT?	Days Weeks Months I have ongoing symptoms
Have you developed any new diseases or symptoms following FMT?	Yes No If yes, describe
Have you noticed improvement in any other medical conditions after FMT?	Yes No If yes, describe
Are you concerned about infection risk from FMT?	Yes No
Are you satisfied with your treatment outcome?	Yes No
Who do you believe would be an ideal donor?	Spouse Sibling Friend or unrelated contact Anonymous screened donor
Do you think a third party (i.e. Medicare, private insurance or state government) should subsidise the costs to patients for recurrent or refractory FMT?	Yes No
In your opinion, should FMT be classified as:	Bodily tissue donation Therapeutic drug Unsure

CDI, *Clostridioides difficile* infection; FMT, fecal microbiota transplantation.

**Table 2:** Physician survey questions

Question	Options
What is your gender	Male Female
What is your age?	18–24 25–34 35–44 45–54 55–64 65+
What is your speciality?	Gastroenterology Infectious diseases
What is the nature of the majority of your practice?	Advanced trainee Staff specialist Private practice physician Visiting medical officer Predominantly medical research
Are you aware of the existence of an FMT service in South Australia?	Yes No
Have you ever referred a patient with CDI for FMT?	Yes No
If the above answer was yes: How many patients? In how many has the treatment been successful? Could you envisage using the service again in the future?	
Have you seen any new diseases develop in your patients following FMT?	No Yes (please specify)
In you patients who have received FMT for CDI and who have other medical comorbidities, have you noticed any improvement or deterioration in these conditions following FMT?	Improvement (please specify) Deterioration (please specify) No change
For which of the following patients with C. difficile in an outpatient setting would you consider FMT? (may select more than one answer)	Prior to antibiotic therapy Immediately following first treatment course of antibiotics Following first recurrence (post antibiotic therapy) Following second recurrence (post antibiotic therapy) Following three of more recurrences (post antibiotic therapy)
For which of the following patients hospitalised with C. difficile would you consider FMT? (may select more than one answer)	Prior to antibiotic therapy Following antibiotic therapy for first episode of CDI Following first recurrence (post antibiotic therapy) Following second recurrence (post antibiotic therapy) Following three of more recurrences (post antibiotic therapy)
For which of the following patients with C difficile would you consider FMT? (may select more than one answer)	Following a severe episode requiring supportive care (HDU or ICU) Patient not responding to antibiotics Other (please specify)
Do you believe most of your patients with recurrent or refractor CDI would consider FMT?	Yes No
If above answer was no—what do you think would be their main reason for not considering FMT?	Aesthetic reasons (i.e. “gross” factor)? Infection risk Other transmissible disease risk
Are you concerned regarding potential alteration in the recipient's microbiome?	No Yes (please explain)
Are you concerned about disease transmission risk?	Yes No
If above answer was yes, what are your main concerns? (may select more than one answer)	Infection Metabolic risk (i.e. obesity, insulin resistance) Autoimmune disease Other (please specify)
Do you believe these risks outweigh the potential benefits?	Yes No
How do you believe FMT should be delivered?	Via colonoscopy Via endoscopy Naso‐jejunal tube Enemas
Who do you believe would be an ideal donor?	Spouse Sibling Friend or unrelated contact Donor anonymous to the recipient
Do you think a third party (i.e. Medicare, private insurance or state government) should subsidise the costs to patients for recurrent or refractory FMT?	Yes No
There is a current debate about the regulation of FMT. In your opinion, should processed donor faeces for FMT be classified as:	Bodily tissue donation Therapeutic drug

CDI, *Clostridioides difficile* infection; FMT, fecal microbiota transplantation; HDU, high dependency unit; ICU, intensive care unit.

### *Clinical data*

The South Australian FMT for CDI database was established in 2013 and prospectively recorded patient demographic details, clinical details of CDI, and outcomes of FMT for CDI. Attempts were made to contact all patients within 3 months following FMT to assess clinical cure. In case a patient could not be contacted, medical records were reviewed. Primary cure was defined as resolution of symptoms or a negative *C. Difficile* toxin test following a single FMT; secondary cure was defined as resolution of symptoms or a negative *C. Difficile* toxin test following multiple FMTs.

## Results

### *Patient survey*

Patient demographics, disease, and treatment characteristics are shown in Table 3. Regarding patient perceptions prior to FMT, 37 of 54 (69%) patients reported having no concerns regarding FMT prior to the procedure. The remaining 17 (31%) patients had reservations when first offered FMT; in all 17 patients, the concerns were aesthetic (the “yuck factor”); 6 of these 17 patients were also concerned about infection, and 2 were concerned about the colonoscopy procedure. Awareness of FMT prior to developing CDI was relatively low, with only 20 (37%) of patients reporting prior knowledge of the procedure. Of the 20 patients under 60 years of age, 10 (50%) had prior awareness, compared with 10 of 34 (29%) of the patients over 60 years of age. Overall, 29 of 54 (54%) first heard of FMT from a medical specialist, 17 (31%) heard about FMT through the media, and only 2 (3.7%) heard through their general practitioner (GP).

**Table 3:** Demographics of patient respondents and disease and treatment characteristics

Total respondents	*n* = 54
Female gender, *n* (%)	36 (67)
Median age, *n* (IQR)	65.5 (51–79)
Route of FMT administration	
Colonoscopy, *n* (%)	51 (94)
Push enteroscopy, *n* (%)	1 (2)
Colonoscopy and enteroscopy, *n* (%)	1 (2)
Enema, *n* (%)	1 (2)
Median number of relapses prior to FMT, *n* (range, IQR)	3 (0–12, 2–4)
Primary cure rate in respondents, *n* (%)	51 (94)
Timing of symptom response to FMT	
Within days, *n* (%)	32 (59)
Within weeks, *n* (%)	13 (24)

FMT, fecal microbiota transplantation; IQR, interquartile range.

Almost all patients (52/54 [96%]) would recommend FMT to others, and 51 of 54 (94%) were satisfied with treatment outcome. Primary cure was achieved in 51 (94%) (compared with 26/31 [84%] of patients who did not return the survey).

The majority of respondents, 40 of 46 (87%), believed the ideal donor was anonymous to the recipient, with 2 (4%) preferring a sibling, 1 (2%) preferring a friend, and 3 (5%) happy with any donor, known or anonymous. When asked how FMT should be regulated, 33 of 54 (61%) thought FMT should be classified as a bodily tissue donation; only 7 (13%) thought FMT should be classified as a drug, and 14 (26%) were unsure. All 54 patients (100%) believed the cost should be covered by government or medical insurance providers.

### *Physician survey*

#### *Respondents*

**Figure 1:**
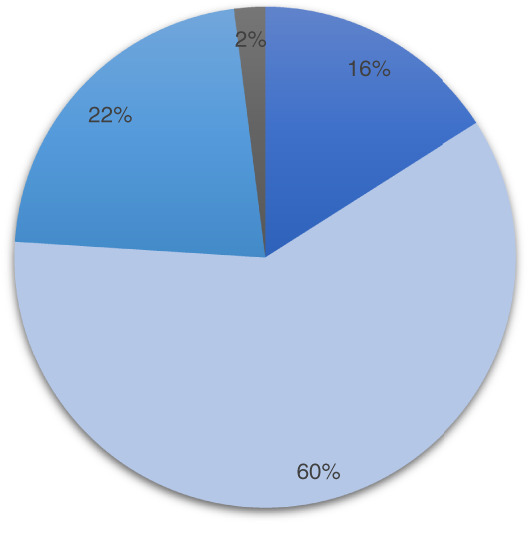
Job descriptions of physician respondents. (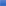), Advanced trainee; (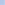), staff specialist; (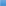), private practice; (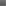), predominantly research.

One hundred physicians were contacted, and 50 completed the online survey (50% response, male: female 32:18). Gastroenterology (GE) consultants or advanced trainees made up 66% (33/50); 17 (34%) were ID consultants or advanced trainees (Fig. 1).

#### *Experience with* *FMT*

The majority of physicians, 48 of 50 (96%), were aware of the existence of the FMT service, and 29 (58%) had referred at least one patient with CDI for FMT (median 2, interquartile range [IQR] 1–2), with 28 of the 29 (97%) witnessing primary cure in their patient(s). All physicians could envisage using the service again in the future. Regarding previous patient referrals, 21 of 33 (64%) of gastroenterologists had prior experience, compared with 8 of 17 (47%) of ID physicians. None noted new diseases developing in their patients following FMT.

#### *Indications for* *FMT*

In the outpatient setting, 57% of clinicians would consider FMT after the second recurrence, and 55% would consider FMT after three or more recurrences (Table 4). In the inpatient setting, this was 61 and 47%, respectively. After the first recurrence in the outpatient setting, 18% of clinicians would consider FMT, compared with 41% in the inpatient setting. Only one clinician (2%) would consider referring first line, that is, prior to antibiotics, in either setting. Following a severe episode of CDI requiring supportive care, 66% would consider FMT upfront, and 96% would consider FMT in a patient not responding to antibiotics.

**Table 4:** Number of physicians, *n* (%), who would refer for fecal microbiota transplantation (FMT) for each indication

Indication	Outpatient	Inpatient
Prior to antibiotics	1 (2%)	1 (2%)
Immediately after first course of antibiotics	1 (2%)	6 (12%)
Following first recurrence	9 (18%)	20 (41%)
Following second recurrence	28 (57%)	30 (61%)
Following ≥3 recurrences	27 (55%)	23 (47%)

#### *Concerns regarding patient perception of* *FMT*

The majority of physicians, 45 of 50 (90%), believed their patients would consider FMT a treatment. Among the small group of physicians who thought that their patients would not consider FMT, all predicted that this would be for aesthetic reasons rather than for risk of infection or disease transmission.

#### *Concerns regarding safety of* *FMT*

**Figure 2:**
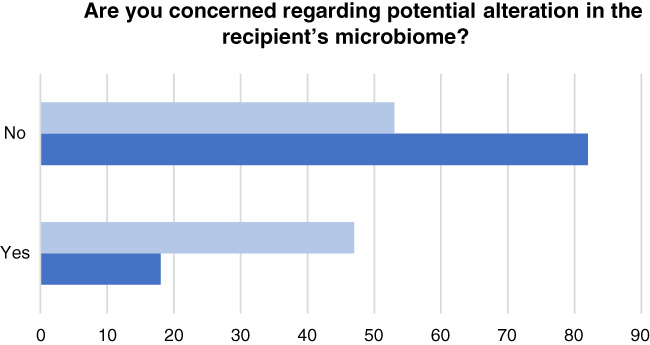
Clinicians (% per specialty) concerned about potential alteration of microbiome in fecal microbiota transplantation recipients. (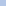), Infectious diseases; (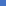), gastroenterology.

A minority of physicians, 14 of 50 (28%), expressed concern regarding potential alteration of the microbiome, as shown in Figure 2. ID physicians had significantly greater concern regarding potential deleterious alteration of the microbiome than gastroenterologists (8/17 [47%] *vs* 6/33 [18%]; *P* = 0.047). Nearly half of physicians, 23 of 50 (46%), were concerned about disease transmission risk; there was no difference between the specialties for this question. Of these physicians, 17 of 23 (76%) were concerned about infection, 12 (57%) were concerned about metabolic risk (e.g. obesity, insulin resistance), and 9 (43%) were concerned about autoimmune disease risk. When asked whether the risk of FMT would outweigh the benefit, 15 of 50 (30%) responded “yes”; however, 8 of these physicians had referred patients for FMT, with primary cure in all but one.

#### *Classification and regulation of* *FMT*

The majority of physicians, 36 of 50 (72%), thought that FMT should be classified as a bodily tissue donation; 12 (24%) thought it should be classified as a drug, and all 50 physicians (100%) believed FMT should be funded by a third party.

## Discussion

These data give insight into the views of patients who have undergone FMT for CDI and physicians who have had access to this therapy for their patients via a stool bank. FMT for CDI has a strong evidence base from multiple randomized controlled trials^3^, ^5^, ^16^ and has been accepted as the standard of care in major international treatment guidelines.^9^, ^11^, ^17^, ^18^ However, evidence of efficacy and safety alone have not been sufficient to facilitate the widespread uptake and availability of FMT therapy. In additional to current regulatory frameworks hampering the availability of FMT, patient and physician views on FMT may have a bearing on prescribing and could diverge from best practice guideline recommendations.^19^, ^20^, ^21^

The results of this survey suggest that perceived risks of FMT by many physicians may be a potential barrier to the use of this therapy. These perceived risks do not appear to be due to physicians' experience with adverse events as none of the physicians surveyed reported noting new diseases developing in their patients following FMT, and most achieved primary cure. Despite very few reported adverse events in many thousands of treated patients in the literature, a concerning 30% of physicians still reported that the potential risks of FMT outweigh the benefit. The findings of this survey are in keeping with previous Australian data: Paramsothy *et al*.,^22^ in a survey of 52 gastroenterologists in 2015, found that over half of the surveyed physicians were concerned about lack of evidence for efficacy, and 15% did not believe FMT was an effective therapy, even in the setting of CDI. Two thirds believed their patients would be concerned by the aesthetic factor despite patients' willingness to consider FMT being well established. Severe or fulminant CDI carries a high mortality, reported to be between 36 and 58%, which is not mitigated by colectomy.^23^, ^24^, ^25^ A recent study of FMT in this setting demonstrated that the number needed to treat with FMT to prevent one death was 3.2 relative to standard antibiotic therapy.^26^ A lack of utilization of FMT, particularly in this context, is life threatening and of great concern.

Almost half (46%) of physicians were concerned about disease transmission risk, and the majority of these concerns centered on infection risk. Much lower proportions of patients shared these concerns, with only 11% concerned about infection risk. Interestingly, this survey was undertaken prior to reports of transmission of antibiotic‐resistant bacteria producing an extended‐spectrum beta‐lactamase (ESBL) via FMT, which may have further raised concern among physicians and patients alike.^27^ A considerable number of physicians also harbored concerns regarding metabolic and autoimmune disease risk, but again, this was not a concern held by most patients in this cohort. ID physicians were particularly concerned about altering the gut microbiota with FMT and associated risks. This is surprising given that ID physicians routinely prescribe and monitor antibiotic prescriptions and therefore may be more aware of the disease associations, with loss of microbiota diversity as a result of antibiotic use.^28^, ^29^, ^30^, ^31^, ^32^ There is, however, far less evidence that the gain in microbial diversity, or change in microbial composition following FMT, results in disease. Although short‐term data are reassuring, larger case control studies and registry data will be required to properly assess long‐term unknown risks of disease transmission via FMT.

In contrast to physicians, patients in this survey reported high rates of acceptance for FMT as a therapy for rCDI. Patients noted rapid improvement in their symptoms following FMT, and adverse events were minor and infrequent. Despite a third of patients having concerns regarding aesthetics prior to the procedure, almost all patients were satisfied with the treatment and would recommend it to others. This is also consistent with previous studies. While patient aversion to the aesthetics of FMT is cited by clinicians as a barrier to its use,^33^ in a large survey of 192 patients, when provided with efficacy data for CDI treatments, 85% of patients chose FMT, with only 4% deterred by its fecal composition.^34^ Furthermore, high symptom burden and morbidity has been shown to be a powerful motivator for acceptance of FMT as a treatment,^35^ and high patient satisfaction has previously been reported.^13^

Awareness of this therapy seems to be increasing over time, with a third of patients in this cohort first hearing of FMT through the media, as opposed to their general practitioner or medical specialist. Increased public and media interest in the procedure may have resulted in increased acceptability as the procedure is normalized. Very few patients had heard of FMT from their GP, and this may reflect a lack of awareness among primary care doctors or that these patients are primarily being treated in the hospital system. Physician experience is also increasing, with 58% of our cohort having referred patients for FMT, which is much higher than previous similar reports, particularly by Paramsothy *et al*.^22^ 4 years ago, where only 21% of the 52 Australian gastroenterologists surveyed had referred a patient for FMT, despite 90% reporting that they would refer patients if FMT was easily available.

The majority (72%) of physicians believed that FMT should be regulated as a tissue product and not a drug, and both patients and physicians wanted FMT to be funded by a third‐party payer. Regulatory uncertainty continues to pose as a barrier for service delivery, and the development of a regulatory framework is essential for the efficient and safe delivery of this therapy.^12^, ^36^

This study had a number of limitations. First, a third of patients did not respond to the survey, and primary cure was more common in those who responded, which may overestimate the reports of satisfaction with treatment. Only half of physicians responded, and there is potential for bias here as those who took the time to respond may be more interested in or more experienced with FMT. Secondly, all physicians were practicing in South Australia, and so, there are potential limitations on the generalizability of the responses. However, given that South Australia has had uninterrupted access to a stool bank since 2013, limiting the survey to this jurisdiction is informative as it removes the important variable of a lack of access to therapy. There was also no standardized method of reporting adverse events in this survey, although global satisfaction was more the focus.

Despite the wealth of evidence supporting the safety and efficacy of FMT, and increasing patient awareness and acceptance as reflected in our survey, physician reservations may still present a barrier to uptake of this therapy, even in a region such as South Australia where access to FMT is readily available through a centralized stool bank. Publication of long‐term data on adverse effects, particularly infection risk and metabolic disease risk, will be important in assuaging these concerns and encouraging adherence to guideline recommendations. The establishment of national FMT registries will be important in this regard. Eliminating the regulatory ambiguity surrounding FMT and allowing for national standardization may also help to improve accessibility and acceptability of this life‐saving therapy.
